# Supplementary material for: Physical and Dietary Intervention with Opuntia ficus-indica (Nopal) in Women with Obesity Improves Health Condition through Gut Microbiota Adjustment
Source: Nutrients. 2022 Feb 27;14(5):1008. doi: 10.3390/nu14051008 (PMC8912383; doi:10.3390/nu14051008)
Supplement: Supplementary file 1 [file nutrients-14-01008-s001.zip › nutrients-1549459-supplementary.pdf]

BioTech

Article

Physical and dietary intervention with *Opuntia ficus-indica* (nopal) in women with obesity improves health condition through gut microbiota adjustment

Karina Corona-Cervantes, Alicia Parra-Carriedo, Fernando Hernández-Quiroz, Noemí Martínez-Castro, Juan Manuel Vélez-Ixta, Diana Guajardo-López, Jaime García-Mena, and César Hernández-Guerrero.

Supplementary Material

Tables

Table S1 Sequencing summary after trimming[#] (n=72).

Parameter		Fecal samples n=72
Length[a]		183 bp (10.82) [b]
Forward sequence counts		7’362,905
	mean	102,263
	min-max	9,150 - 667,584
	Samples with <10,000 sequence counts	1
Feature counts		6’150,471
	count mean	85,423.21
	count min–max	7,461-493,759
	Samples with <10,000 feature counts	1

[#]Reads were analyzed using FastQC software v0.11.9 and trimmed at 200 nt with Trimmomatic v0.38, for make a denoising with q2-dada2=2021.2.0. [a]Length expressed as bases; [b]Standard deviation.

Table S2 Relative abundance of phyla in fecal samples during nopal diet intervention.

Taxa	Beginning	Ending	*p- value*
**Normal weight group**			
Firmicutes (%)	48.48 ± 0.176	67.84 ± .174	0.130
Bacteroidetes (%)	47.16 ± 0.179	27.68 ± 0.174	0.168
Proteobacteria (%)	2.04 ± 0.031	2.13 ± 0.034	0.858
Actinobacteria (%)	2.18 ± 0.034	1.58 ± 0.025	0.325
Cyanobacteria (%)	0.06 ± 0.009	0.09 ± 0.003	0.655
**Obesity group**			
Firmicutes (%)	71.39 ± 0.151	70.42 ± 0.133	0.737
Bacteroidetes (%)	24.72 ± 0.136	26.60 ± 0.122	0.518
Proteobacteria (%)	2.13 ± 0.026	1.56 ± 0.017	0.829
Actinobacteria (%)	1.58 ± 0.028	1.26 ± 0.013	0.665
Cyanobacteria (%)	0.09 ± 0.002	0.05 ± 0.000	0.317

Data are mean ± standard deviation as indicated (see Fig. 1). The p-values were calculated using Wilcoxon signed-rank test. *p* values <0.05 are considered statistically significant. Relative abundance for "Other" phyla not shown in the table, corresponds to 0.09% for Beginning Normal Weight group, 0.68% for Ending Normal Weight group, 0.1% for Beginning Obesity group and 0.1% for Ending Obesity group.

Table S3 Relative abundance of taxa in fecal samples during nopal diet intervention.

Taxa	Beginning	Ending	*p- value*
**Normal weight group**			
Bacteroides	0.273 ± 0.189	0.208 ± 0.171	0.47
Ruminococcaceae	0.13 ± 0.095	0.119 ± 0.073	0.77
Lachnospiraceae	0.085 ± 0.056	0.093 ± 0.056	0.64
Ruminococcaceae Ruminococcus	0.066 ± 0.062	0.025 ± 0.014	0.03
Blautia	0.04 ± 0.023	0.074 ± 0.065	0.37
Prevotella	0.031 ± 0.042	0.013 ± 0.019	0.28
Streptococcus	0.026 ± 0.036	0.022 ± 0.019	1.00
Barnesiellaceae	0.019 ± 0.017	0.01 ± 0.008	0.28
Eubacterium	0.003 ± 0.004	0.057 ± 0.165	0.83
Clostridiales	0.045 ± 0.019	0.052 ± 0.032	0.58
Erysipelotrichaceae	0.014 ± 0.015	0.047 ± 0.06	0.02
Lachnospiraceae Ruminococcus	0.01 ± 0.011	0.01 ± 0.013	0.83
Rikenellaceae	0.048 ± 0.038	0.032 ± 0.029	0.52
Clostridiaceae	0.003 ± 0.003	0.007 ± 0.011	0.70
Roseburia	0.009 ± 0.013	0.021 ± 0.025	0.16

	**Obesity group**		
Bacteroides	0.099 ± 0.118	0.088 ± 0.097	0.58
Ruminococcaceae	0.137 ± 0.075	0.13 ± 0.046	0.83
Lachnospiraceae	0.154 ± 0.058	0.161 ± 0.044	0.6
Ruminococcaceae Ruminococcus	0.034 ± 0.023	0.024 ± 0.013	0.05
Blautia	0.085 ± 0.051	0.08 ± 0.031	0.83
Prevotella	0.088 ± 0.119	0.111 ± 0.132	0.3
Streptococcus	0.007 ± 0.013	0.008 ± 0.013	0.54
Barnesiellaceae	0.007 ± 0.009	0.008 ± 0.008	0.63
Eubacterium	0.002 ± 0.008	0.004 ± 0.011	0.48
Clostridiales	0.059 ± 0.032	0.059 ± 0.026	0.75
Erysipelotrichaceae	0.003 ± 0.005	0.007 ± 0.017	0.37
Lachnospiraceae Ruminococcus	0.055 ± 0.054	0.042 ± 0.067	0.35
Rikenellaceae	0.028 ± 0.032	0.028 ± 0.043	0.56
Clostridiaceae	0.024 ± 0.029	0.025 ± 0.056	0.51
Roseburia	0.018 ± 0.028	0.03 ± 0.041	0.54

Data are mean ± standard deviation as indicated. The *p-values* were calculated using Wilcoxon signed-rank test. *p* values <0.05 are considered statistically significant.

Table S4 Alfa diversity indexes of bacterial microbiota during nopal diet intervention.

Taxa	Beginning	Ending	*p*- value
	**Normal weight group**		
Observed	237.00 ± 80.021	220.00 ± 58.902	0.790
Chao1	237.00 ± 80.021	220.00 ± 58.902	0.790
Shannon	3.99 ± 0.350	3.99 ± 0.341	0.965
Simpson	0.96 ± 0.020	0.96 ± 0.016	1.000
	**Obesity group**		
Observed	175.20 ± 56.445	192.52 ± 72.801	0.383
Chao1	175.20 ± 56.445	192.52 ± 72.801	0.383
Shannon	3.81 ± 0.569	3.76 ± 0.256	0.427
Simpson	0.94 ± 0.065	0.95 ± 0.019	0.693

Data are mean ± standard deviation as indicated (see Fig. 2). The p-values were calculated using Wilcoxon signed-rank test. *p* values <0.05 are considered statistically significant.

Figures

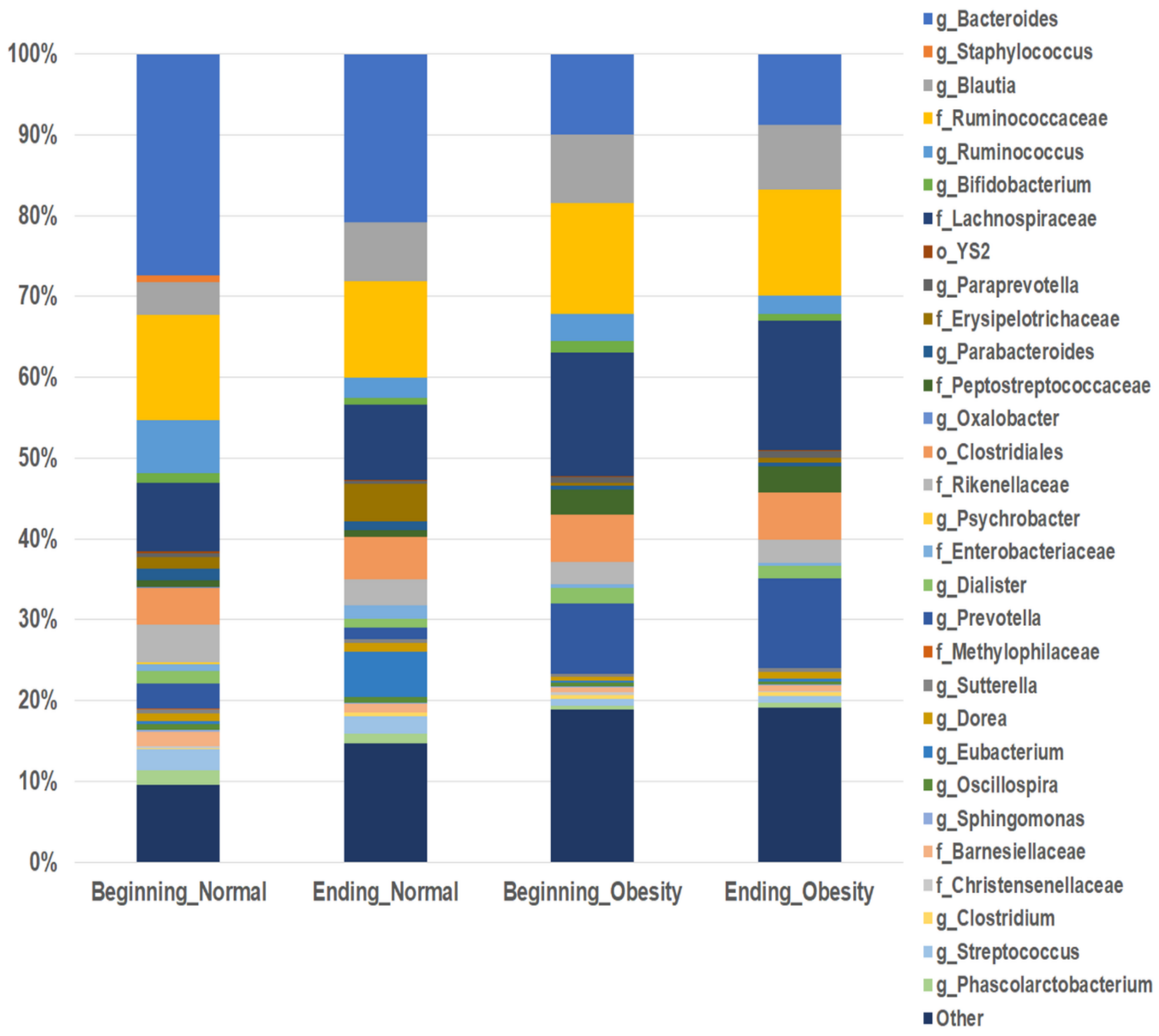

Figure S1. Relative abundance of bacterial order, family, and genus in fecal samples during nopal diet intervention. Figure shows data for Beginning normal weight, Ending normal weight, Beginning obesity, Ending obesity. Sectors in bar plots indicates main taxa as shown by tag colors at the right of the figure. Abundances of each bacterial taxa are shown as percentage in the Y-axis, and diet group in the X-axis. "Other" groups taxa with < 0.50 % relative abundance.

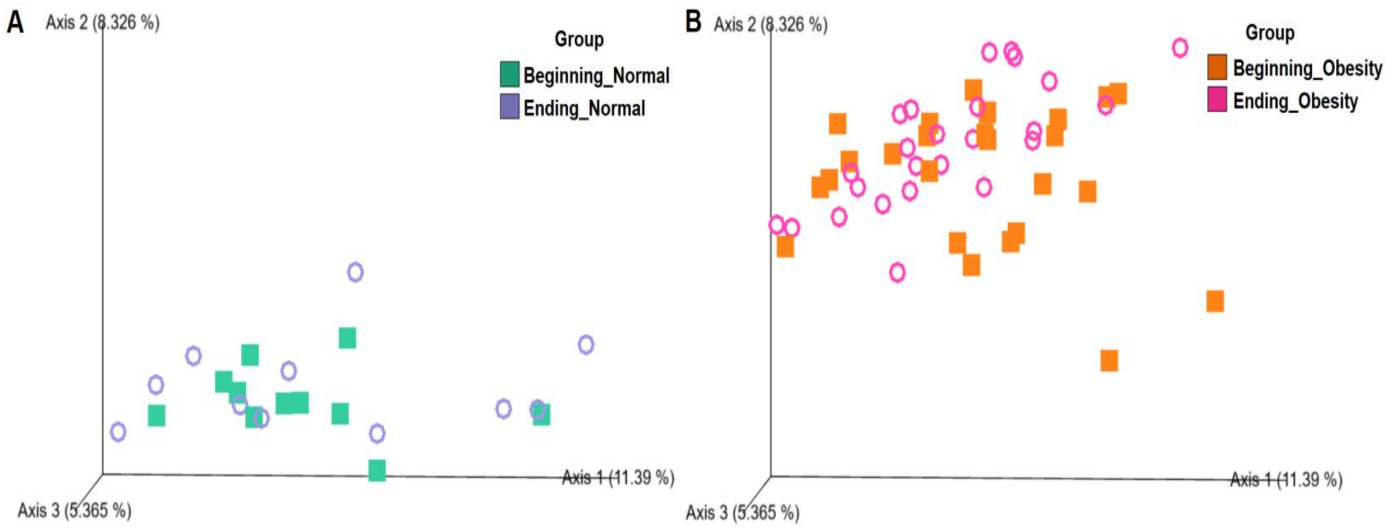

Figure S2. Beta diversity of bacteria in fecal samples during nopal diet intervention. The graphics show beta-diversity analyses calculated by dissimilarity metrics using OTU tables and Unweighted UniFrac analyses. The analyses show the dissimilarity among groups Normal weight at beginning (green square), and ending (blue ring), Obesity at beginning (orange square), and ending (pink ring). The three-dimensional scatter plots were generated using principal coordinates analyses (PCoA) in three different axes which shows the percentage of total differences.

---end-of-document—
